# Brain–Heart Axis: Brain-Derived Neurotrophic Factor and Cardiovascular Disease—A Review of Systematic Reviews

**DOI:** 10.3390/life13122252

**Published:** 2023-11-25

**Authors:** Massimo Fioranelli, Maria Luisa Garo, Maria Grazia Roccia, Bianca Prizbelek, Francesca Romana Sconci

**Affiliations:** 1 Department of Human Sciences, Guglielmo Marconi University, 00193 Rome, Italy; m.fioranelli@unimarconi.it (M.F.); m.roccia@unimarconi.it (M.G.R.); 2 Istituto Terapie Sistemiche Integrate, Casa di Cura Sanatrix, 00199 Rome, Italy; bianca.przybylek@goglemail.com (B.P.); francescasconci@libero.it (F.R.S.)

**Keywords:** BDNF, brain-derived neurotrophic factor, cardiovascular disease, Val66Met, polymorphism, stroke, heart failure, angina

## Abstract

Background: The brain–heart axis is an intra- and bidirectional complex that links central nervous system dysfunction and cardiac dysfunction. In recent decades, brain-derived neurotrophic factor (BDNF) has emerged as a strategic molecule involved in both brain and cardiovascular disease (CVD). This systematic review of systematic reviews aimed to (1) identify and summarize the evidence for the BDNF genotype and BDNF concentration in CVD risk assessment, (2) evaluate the evidence for the use of BDNF as a biomarker of CVD recovery, and (3) evaluate rehabilitation approaches that can restore BDNF concentration. Methods: A comprehensive search strategy was developed using PRISMA. The risk of bias was assessed via ROBIS. Results: Seven studies were identified, most of which aimed to evaluate the role of BDNF in stroke patients. Only two systematic reviews examined the association of BDNF concentration and polymorphism in CVDs other than stroke. Conclusions: The overall evidence showed that BDNF plays a fundamental role in assessing the risk of CVD occurrence, because lower BDNF concentrations and rs6265 polymorphism are often associated with CVD. Nevertheless, much work remains to be carried out in current research to investigate how BDNF is modulated in different cardiovascular diseases and in different populations.

## 1. Introduction

Cardiovascular disease (CVD) is a leading cause of death and disability worldwide, affecting approximately 523 million people in the world [1]. The World Health Organization estimates that the number of deaths due to cardiovascular disease is about 17.9 million/year [2], and this number is expected to increase to 24 million by 2030 because of the aging population and the increase in some risk factors, such as air pollution and climate change [1]. Data over the past 30 years show a marked increase in chronic diseases, particularly in higher-income countries, due to a number of reasons, including improvements in surgical and pharmacological treatments and lifestyle changes that have reduced mortality, especially in high-income countries [3].

The recent Global Burden of Diseases, Injuries, and Risk Factors (GBD) study tracking trends in death and disability since 1990 identified several nongenetic risk factors for cardiovascular disease, ranging from hypertension, high LDL-C, and poor lifestyle habits (e.g., poor diet, smoking, alcohol consumption, and a lack of physical activity) to environmental risks such as air pollution or suboptimal temperatures [4].

In recent decades, an increased interest in studying important biomarkers for evaluating genetic susceptibility or responses to a CVD event has emerged [5,6]. Among several proposed biomarkers for CVD, the literature has focused on evaluating the role of brain-derived neurotrophic factor (BDNF) [7,8,9], a neurotrophic growth factor belonging to the family of neurotrophic factors that is responsible for the differentiation, maturation, and survival of neurons in the nervous system, as well as for regeneration processes, the regulation and remodeling of synapses, and the control of plasticity [10,11,12] through its high-affinity receptor tropomyosin receptor kinase B (TrkB) [13]. BDNF is synthesized in the endoplasmic reticulum as a 32–35 kDa precursor protein known as proBDNF that migrates through the Golgi apparatus and the trans-Golgi network. In the presence of the sorting receptor carboxypeptidase E, which is associated with lipid rafts, proBDNF is sorted by vesicles and subsequently transported for activity-dependent secretion by postsynaptic dendrites. Subsequently, proBDNF is cleaved by a specific protein convertase enzyme to form 13 kDa biologically active mature BDNF (mBDNF), which, in turn, binds with higher affinity to TrkB [13].

The half-life of BDNF in the brain is not known; due to its similarity to nerve growth factor, its half-life is estimated to be less than one hour [14], while circulating BDNF has been shown to have a half-life of less than 10 min [15].

BDNF is produced in neurons and glial cells and is widely distributed in various regions of the brain (e.g., in the cortex, hippocampus, and cerebellum). Astrocytes, the most abundant cell type in the mammalian brain and also the largest type of glial cell, are important sources of BDNF. Under normal conditions, BDNF released from astrocytes has neuroprotective and neuroregenerative effects. Furthermore, astrocytes stimulate microglia to express BDNF by releasing cytokines that trigger microglial remodeling and regulate their function [16]. BDNF is also distributed in the lungs, heart, spleen, gastrointestinal tract, and liver. It is also expressed in fibroblasts, vascular smooth muscle cells, and the thymus stroma [17].

BDNF levels decline across an individual’s lifespan, passing from 9230 pg/mL in infants to 21 pg/mL in the elderly (65–97 years) with a decrease of 1 pg/mL per year. Several factors, such as acute responses to environmental passive agents, hypoxic stress, sun exposure and season, environmental enrichment, physical activity, intermittent fasting or strict caloric restriction, or a ketogenic diet (high-protein, high-fat, low-carbohydrate foods), modulate the release of BDNF [18]. In various pathological conditions, glial cell-derived BDNF is involved in disease recovery and injury repair [16]. In stroke patients, under normal conditions, BDNF levels increase as an adaptive response to the stroke itself to reduce neuronal loss and promote neurogenesis; BDNF released by astrocytes plays a neuroprotective role by regulating signaling pathways, releasing cytokines, and inhibiting apoptosis [19], and is beneficial for axonal myelination and neuronal function [20]. In some cases, BDNF levels decrease, likely due to a downstream induction of BDNF that is the result of altered neuronal excitability with downstream signaling of excitatory neurotransmitters [21]. Animal studies have shown that BDNF expression after myocardial infarction is upregulated by neuronal signaling from the heart to protect the myocardium from ischemic damage and thus exhibit a protective effect against cardiac remodeling [22,23]. Altered BDNF levels have also been observed in neurodegenerative diseases. For example, elevated BDNF levels have been found in patients with early-stage Alzheimer’s disease, probably as a compensatory mechanism to combat early neurodegeneration or to activate immune cells [24]. Up-regulation of BDNF has been observed immediately after post-experimental traumatic brain injury in support of neuroprotection [25,26]. In clinical studies, BDNF levels are negatively correlated with injury severity after traumatic brain injury [27].

The BDNF concentration in peripheral blood is often used to estimate the BDNF concentration in the brain, as BDNF can cross the blood–brain barrier in both directions [28]. Serum BDNF concentration is about 100 times higher and is a more accurate biomarker than plasma, as sample handling and preparation procedures can interfere less [29]. Comparable results have also been demonstrated between serum BDNF levels and whole blood, although the cell lysis step in sample preparation can lead to additional variation as the whole blood must be lysed prior to BDNF measurement [29]. Overall, serum BDNF measurement is thought to more accurately reflect the totality of circulating BDNF, although the standard method to be used has not yet been evaluated [30].

In healthy individuals, serum BDNF levels appear to correlate positively with BDNF levels in the brain and cerebrospinal fluid when BDNF is released from the brain into the blood; however, in older adults, blood BDNF measurements are not representative of BDNF levels in the cerebrospinal fluid [31]. Studies comparing cerebrospinal fluid and serum BDNF measurements in a sample of patients with Alzheimer’s disease found that they do not correlate [32] and that BDNF concentrations in serum or plasma are much higher than in cerebrospinal fluid, possibly due to peripheral synthesis [33]. After severe traumatic brain injury, a negative correlation between cerebrospinal fluid and serum was found, probably due to platelet activation in response to the injured tissue [31].

The study by Lommatzsch et al. (2005) showed that BDNF concentrations were higher in the urinary bladder, lung, and colon than in the brain or skin [34]. Recently, Olivas-Martinez (2023) developed and validated a method to quantify BDNF protein levels in urine samples using different experimental conditions and non-competitive commercial enzyme-linked immunosorbent assay (ELISA) kits. Using urine samples from 256 male adolescents, both upon waking (*n* = 150) and before bedtime (*n* = 106), they demonstrated the adequate sensitivity of their method, which yielded a limit of detection of 0.047 ng/mL, with only 2% of urine samples below this limit. Furthermore, they demonstrated that BDNF concentrations in the urine samples were within the range already reported in the literature, with levels being higher after waking (median = 3.747 ng/mL) than before sleep (median = 1.495 ng/mL) [35]. The ELISA techniques commonly used to quantify BDNF in biological samples [36] are simple and inexpensive, have high specificity and sensitivity, and allow a large number of samples to be analyzed simultaneously; nevertheless, some shortcomings have been noted in some ELISA kits, as they are unable to selectively distinguish between mature BDNF and proBDNF [35].

According to the “brain–heart axis” hypothesis, the brain and heart are characterized by a complex inter- and bi-directional relationship in which mechanisms such as the hypothalamic–pituitary–adrenal axis, the autonomic nervous system, or systemic inflammation link central nervous system (CNS) disorders and cardiac dysfunction [37,38].

BDNF may play a relevant role in explaining this brain–heart axis, as it is involved in a number of brain functions in the hippocampus, cortex, amygdala, striatum, and hypothalamus [39], as well as in several cardiovascular functions, given that circulating BDNF promotes angiogenesis in the vascular system by regulating reactive oxygen species and is expressed and found throughout the cardiovascular system [40,41]. Indeed, since BDNF and its signaling support brain health and neuroplasticity [42] and help neurons cope with stressful conditions, they also support normal cardiac muscle in adults [22,43]. Blood BDNF levels depend on the activity-dependent release of BDNF from hypothalamic neurons [39]. This means that BDNF released from neurons can not only affect the brain locally by modulating neural network strength, but can also affect other organs distant from the brain via the bloodstream [39].

The etiology of cardiovascular disease is atherosclerosis, a complex chronic inflammatory process initiated by activation of the endothelium and followed by a cascade of events resulting in vasoconstriction and the activation of inflammatory processes, leading to the formation of atheroma plaque [44]. Decreased BDNF in the blood may cause endothelial and vascular dysfunction and atherosclerosis due to increased oxidative stress and inflammation [40,45].

In stroke patients, BDNF release has been reported as a positive prognostic factor for patients poststroke recovery so that increasing BDNF levels are associated with faster and stronger recovery, while low levels of BDNF are associated with high stroke risk and poor recovery [46]. Okada et al. (2012) demonstrated that the deletion of BDNF or disruption of TrkB in the heart leads to worsening of cardiac dysfunction after myocardial infarction [23].

To date, several studies and reviews have shown that altered BDNF levels are common in patients with cardiovascular diseases and that specific BDNF genotypes (e.g., Val66Met) are common in patients at higher risk for cardiovascular disease. However, the significant number of studies reported on the association between BDNF and cardiovascular disease and the large heterogeneity of these studies due to specific contexts (e.g., ethnicity), designs (e.g., time of BDNF measurement detection), and patient characteristics (e.g., age or CVD severity) prevent the current literature from providing an overview that would enable weighting of the precise role of BDNF in the context of cardiovascular disease prevention and treatment and directing scientific research toward more standardized studies.

Therefore, to address this issue, we conducted a systematic review of systematic reviews, which is aimed at (1) identifying and summarizing the evidence on the role of the BDNF genotype and BDNF concentrations (i.e., serum or plasma) in assessing CVD risk, (2) assessing the evidence on the use of BDNF as a biomarker for CVD recovery, and (3) evaluating rehabilitation approaches capable of restoring BDNF concentrations.

## 2. Materials and Methods

A systematic review was conducted using the methods described by Smith et al. (2011) [47]. The Preferred Reporting Items for Systematic Reviews and Meta-Analyses statement was applied [48].

### 2.1. Eligibility Criteria

We included systematic reviews or integrative reviews conducted using PRISMA or MOOSE guidelines that included studies involving subjects with CVD, which assessed BDNF concentrations (serum or plasma) or BDNF polymorphisms in CVD subjects. Reviews were included if they used a search strategy in at least two literature databases, regardless of language, publication date, and the study design of primary studies; used a defined article selection procedure; and examined BDNF gene polymorphisms or evaluated the effect of rehabilitation on BDNF levels. Narrative or literature reviews and reviews not included in the definition of a systematic review or an integrative review were excluded. Systematic reviews investigating multiple genetic variants that are different from BDNF were also excluded.

### 2.2. Outcomes

The main outcome of interest was the evaluation of BDNF concentrations or polymorphisms in CVD patients.

### 2.3. Search Strategy

A thorough search strategy was conducted in PubMed, Scopus, Web Of Science, Cochrane Database of Systematic Reviews, and Science Citation Index up to September 2023 using the following keywords: BDNF, brain-derived neurotrophic factor, pro-BDNF, mature BDNF, cardiovascular disease, coronary disease, heart failure, heart disease, atrial fibrillation, myocardial infarction. A hand-searching strategy was also implemented in Google Scholar and in relevant peer-reviewed journals that published any review about the role of BDNF on cardiovascular disease. Furthermore, hand-searching was implemented in published narrative or literature reviews investigating BDNF concentrations or polymorphisms and CVD.

After the complete list of articles was uploaded on Rayyan (https://www.rayyan.ai), articles were evaluated by title and abstract. Then, the articles that were considered for inclusion were retrieved and evaluated by reading the full texts. Then, the two reviewers who had already made the previous selection (MGR and MLG) read the full texts and assessed the eligibility of each study. Finally, the other two reviewers (MF and MLG) reviewed all included and excluded articles and resolved disagreements among reviewers. The reasons for the exclusion of each article were recorded. Disagreements between the reviewers were resolved through consensus among all reviewers.

### 2.4. Data Extraction

Two reviewers (MGR and BP) independently extracted data and reported them in a datasheet (Excel file). The collected data were as follows: (1) year, (2) last data update, (3) cardiovascular disease investigated, (4) database, (5) PROSPERO registration, (6) guidelines, (7) eligibility criteria of included studies, (8) number of included studies, (9) total number of included patients, (10) outcome, (11) assessment of methodological quality, (12) GRADE, (13) meta-analysis, (14) main results, (15) overall conclusion.

### 2.5. Quality Assessment

Quality assessment was performed using the ROBIS tool (citation). ROBIS was composed of a series of 25 items divided according to the following topics: study eligibility criteria (Domain 1), identification and selection of studies (Domain 2), data collection and study appraisal (Domain 3), and synthesis and findings (Domain 4). Finally, a fifth session, called Risk of Bias in the Review, was fulfilled for assessing risk of bias related to the interpretation of findings, relevance of identified studies in address the review’s research question, and whether the reviewers avoided emphasizing results on the basis of their statistical significance. A final evaluation was conducted for each review according to the three difference degrees of bias (low, unclear, high). Two reviewers assessed review quality (MLG and MGR). For studies retrieved in pre-print version, methods of the peer-review process were applied. Discrepancies were resolved via discussion.

### 2.6. Data Synthesis

After identifying the CVD events reported in each of the included studies, a separate synthesis was performed for stroke and other CVDs, respectively, according to the number of retrieved materials. A narrative synthesis was prepared, including meta-analysis results where available. Tables containing relevant information and conclusions were included.

## 3. Results

**Figure 1:**
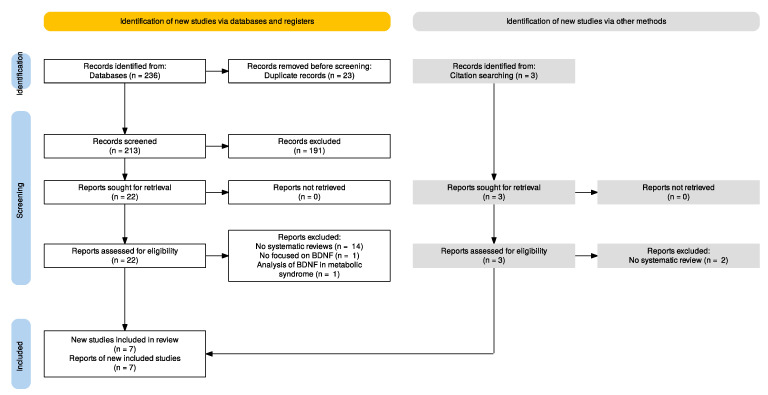
PRISMA flowchart.

By applying the planned search strategy, we retrieved 236 articles from electronic databases. An additional three articles were found through hand-searching (Figure 1). After excluding 23 duplicates and selecting appropriate articles based on title and abstract, 25 articles met the inclusion criteria and were reviewed by reading the full text. Sixteen articles were excluded by reading the full text: sixteen because they were not systematic reviews, one because it was not specific to BDNF, and another because it analyzed BDNF in patients with metabolic syndrome. Finally, seven studies published between 2018 and 2022 were included [49,50,51,52,53,54,55], one of which was retrieved in a pre-print version [55].

### 3.1. Review Characteristics

Six systematic reviews [49,50,52,53,54,55] and one integrative review [51] published between 2018 and 2022 met the eligibility criteria. Table 1 shows a summary of the review characteristics. Six studies examined the role of BDNF in patients with stroke [49,50,51,52,53], including two studies that also examined changes in BDNF in the context of rehabilitation [49,54]. Two studies examined the role of BDNF in specific cardiovascular diseases, focusing specifically on BDNF concentration and/or polymorphisms as risk factors for occurrence and prognosis in myocarditis, heart failure, angina, and Chagas heart disease [51,55]. Most of the reviews included observational studies, mainly case–control studies and cross-sectional studies. Two reviews included randomized controlled trials (RCTs) [49,52]. The risk of bias assessment varied among studies: RCTs were assessed using RoB2; the risk of bias for observational studies was assessed using different approaches. One study used a predefined scale provided by Jiang et al. (2011) that assigns scores based on traditional epidemiological considerations and CVD genetic issues [56]. The QUADAS-2 tool was used in only one study aiming to assess a relationship between the altered level of BDNF and stroke [54]. The GRADE level of evidence was applied in only one study [49]. Five reviews explicitly stated a reference to the PRISMA guidelines [49,50,51,52,55], and one review was conducted according to the MOOSE guidelines [53]. Only one review did not indicate a reference to a guideline used for systematic reviews [54]. Six out of seven studies were meta-analyses (see Supplementary Materials for search strategy—Table S1—and meta-analysis details—Table S2).

### 3.2. Risk of Bias

According to the ROBIS results, only two reviews were rated as having a low risk of bias [49,54]. Two reviews were assessed as having an unclear risk of bias: one was the integrative review, which had some potential sources of bias related to study eligibility criteria, and the other was Shobeiri et al. (2022), which not only had some concerns about study eligibility criteria, but also some concerns about synthesis and outcomes related to not addressing bias in primary studies in the synthesis. The remaining three studies were rated as having a high risk of bias because of concerns about study eligibility criteria, study identification or selection methods (i.e., limitations due to language and no efforts to minimize errors in study selection), data collection, and synthesis and outcomes (i.e., unclear preplanned analyses, a lack of robust evidence, or an inadequate analysis of sources of heterogeneity). Only one review [49] was registered at PROSPERO. Full details of risk of bias are provided in Table 2.

### 3.3. Association between BDNF and CVD

Two systematic reviews investigated a possible role of BDNF in predicting CVD risk and prognosis (Table 3). In the study by Halloway et al. (2020), which included 35 studies (1 controlled intervention, 1 before–after study with no control group, 13 case–control studies, 20 observational cohort and cross-sectional studies), the authors demonstrated that (1) patients with heart failure had lower BDNF levels compared with control subjects, and (2) patients with unstable angina and myocardial infarction had higher BDNF levels. Furthermore, in healthy patients, CVD risk factors such as BMI or higher blood pressure were associated with higher BDNF levels. The results regarding BDNF genotype were variable in relation to race and ethnicity and cardiovascular disease [51].

In the systematic review by Shobeiri et al. (2022), which aimed to summarize the existing evidence assessing BDNF serum and plasma levels in patients with ischemic heart disease and included nine cross-sectional studies, the authors demonstrated, after the exclusion of two outliers, that BDNF levels were significantly lower in patients with ischemic heart disease than in healthy patients (SMD = −0.57, 95% CI: −1.18; 0.04, *p*-value = 0.068, I_2_= 97.2%, *p* < 0.0001). Such significant heterogeneity was probably due to the different approaches to BDNF measurements (plasma or serum). Nevertheless, the specific subgroup analysis to address this issue reported no significant difference between studies using serum or plasma BDNF levels (*p*-value = 0.538). Lower BDNF levels were confirmed in ischemic heart disease in both subgroups (serum: SMD = −0.73, 95% CI: −1.84; 0.37, *p*-value = 0.192; plasma: SMD: −0.37, 95% CI: −0.67; −0.08, *p*-value = 0.013) [55].

### 3.4. Association between BDNF and Stroke

Five systematic reviews [49,50,52,53,54] and the already cited integrative review [51] investigated the association between BDNF genotype and stroke (Table 4).

In a comprehensive systematic review and meta-analysis that included seven case–control studies (cases: 1287, controls: 1032) and aimed to provide a comprehensive evaluation of the relationship between BDNF rs6265 (also known as Val66Met) and ischemic stroke, Bao et al. showed that (1) ischemic stroke risk was lower for the GG genotype in homozygous and dominant models, respectively, than in other models (GG versus AA, OR = 0.57, 95% CI = 0.43–0.75, *p* < 0.0001; GG versus GA + AA, OR = 0.80, 95% CI = 0.65–0.98, *p* = 0.03), and (2) Asian populations, with the exception of Iranians, had a lower predisposition to ischemic stroke in all genetic models, except the heterozygous variant (GG versus AA, OR = 0.58, 95% CI = 0.43–0.77, *p* = 0.0002; GG versus GA + AA, OR = 0.78, 95% CI = 0.62–0.97, *p* = 0.03; G versus A, OR = 0.77, 95% CI = 0.67–0.89, *p* = 0.0003). Contrary to Asian and Caucasian populations, Iranians showed more A alleles than G alleles and a higher risk of ischemic stroke for subjects with G alleles [50].

This different distribution of BDNF alleles was confirmed by Liu et al. (2021), who aimed to evaluate the Val66Met polymorphism distribution through a systematic analysis of 15 longitudinal studies, also evaluating the effects of different genotypes on functional recovery. They showed that Caucasians have fewer A alleles compared with Asian patients (Caucasians: 29.8%, 95% CI: 7.5–52.1%, I_2_ = 99.1%; Asians: 48.6%, 95% CI: 45.8–51.4%, I_2_ = 54.2%) as well as compared to Iranian patients (Caucasians: 18.7%, 95% CI: 16.6–20.9%, I_2_ = 0.00%; Iranians: 87.9%, 95% CI: 83.4–92.3%). For functional recovery, they proved that genetic factors may be partially responsible for the variability in functional recovery in stroke patients, as stroke patients with AA may have worse recovery outcomes than those with GA + GG (OR = 1.90; 95% CI: 1.17–3.10; *p* = 0.010; I_2_ = 69.2%) [53].

Ashcroft et al. (2022), who included 17 studies (6 RCTs, 1 pseudorandomized trial, and 10 nonrandomized studies) to determine the intensity and duration of exercise required to increase BDNF concentrations after stroke, reported a lower risk of ischemic stroke for the GG genotype in both homozygous and dominant models, as shown in the results of impaired BDNF secretion for the A allele (met-BDNF). Furthermore, the same authors showed that a single session of low- or moderate-intensity aerobic exercise or a program of low- or moderate-intensity aerobic exercise did not contribute to a significant increase in BDNF. A significant increase in BDNF favoring physical rehabilitation was obtained with a single session or a program of high-intensity aerobic exercise (single session: MD: 2.49, 95% CI: 1.10–3.88, *p* = 0.001, I_2_ = 0%; aerobic exercise program: MD: 3.42, 95% CI: 1.92–4.92, *p* = 0.00, I_2_ = 2%) [49].

The study by Mojatabavi et al., which included 62 case–control studies (as of August 2021) and examined the relationship between altered BDNF concentrations and stroke, indicated that stroke patients had lower serum BDNF levels than healthy controls (SMD = −1.02, 95% CI: −1.47 to −0.57, *p*-value < 0.001, I_2_ = 96%, *p*-value < 0.001), even excluding influential studies (SMD = −0.92, 95% CI: −1.35 to −0.50, *p*-value < 0.001, I_2_ = 96%, *p*-value < 0.001). The large heterogeneity revealed by the meta-analysis was partially explained by the different age ranges of patients in the included studies (correlation coefficient = −0.11, R_2_ = 62.81%, *p*-value = 0.000). Moreover, the authors showed that the positive effect of physical exercise on BDNF was demonstrated in the period just after the training (SMD = 0.49, 95% CI: 0.09 to 0.88, *p*-value = 0.02, I_2_ = 85%, *p*-value < 0.001), while it was not maintained over a longer period of time. Indeed, no significant effect of the intervention was proved in a delayed phase (SMD = 0.02, 95% CI: −0.43 to 0.47, I_2_ = 83%) [54].

In the 2021 study by Karantali et al. (26 RCTs), to investigate whether serum BDNF levels could be an optimal biomarker for predicting functional outcomes in acute stroke, the authors showed that acute stroke patients had significantly lower serum BDNF levels compared with healthy patients (overall analysis: SMD: −2.37, 95% CI: −4.36, −0.38; sensitivity analysis: SMD: −5.91, 95% CI: −8.40; −3.41, *p* < 0.0001, t_2_ = 10.55, I_2_ = 98.8%) and that the role of BDNF on functional outcome prediction in stroke patients is unclear [52].

The integrative review by Halloway et al. (2020), which found only two studies for the examined association, established that lower serum BDNF levels were associated with poorer functional status 90 days after stroke and higher risk of poor outcomes at 2 and 7 years [51].

## 4. Discussion

Seven systematic reviews on the role of BDNF in CVD published between 2018 and 2022 were retrieved and included in this work.

This work aimed to evaluate the overall evidence that has emerged about the association between BDNF and cardiovascular disease. Overall, from our findings, a clear association emerged between low levels of BDNF, stroke, and heart failure. In terms of methodological approach, the overall evidence appears to be characterized by great heterogeneity given a large number of study designs and methodological approaches in BDNF concentrations measurements. Although six out of seven studies were expressly reported as systematic reviews and meta-analyses, the methodological approaches followed by each of them appear inhomogeneous.

### 4.1. BDNF and Cardiovascular Disease

BDNF is expressed in several cells associated with the cardiovascular system (e.g., endothelial cells, vascular smooth muscle cells, atherosclerotic vessels, etc.) and is involved in oxidative stress in coronary vessels and in the formation of atherosclerotic plaques [57]. The 2015 Framingham Heart Study suggests that high BDNF levels are associated with a lower risk of cardiovascular disease and mortality [58].

In our work, two systematic reviews with a total of 44 studies examined BDNF levels and/or polymorphisms in relation to CVD risk. Lower BDNF levels were observed in patients with heart failure compared with healthy controls, especially in patients with a history of severe cardiovascular disease. The mechanism of action of BDNF in increasing CVD risk could be explained by the association between lower BDNF levels and known CVD risk factors such as lipid levels, older age, male gender, smoking, and diabetes mellitus [59]. Furthermore, altered BDNF levels are involved in endothelial cell dysfunction, a key early process in atherosclerosis development. As demonstrated by Jin et al. (2018), endothelial dysfunction, as measured using von Willebrand factor levels, is associated with decreased circulating BDNF levels and may be predictive of adverse cardiovascular events in patients with coronary artery disease after 12 months [59]. This association could be the result of endothelial injury, which decreases the release of BDNF from endothelial cells into the circulation, likely due to a mechanism in which decreased BDNF levels decrease endothelial cell survival and impair angiogenesis [59]. According to this hypothesis, endothelial dysfunction and low BDNF levels could be considered the cause and effect of each other.

Regarding the roles of the BDNF genotype and cardiovascular diseases, few can be concluded; as already emerged in Halloway et al. (2020), studies conducted using similar populations and study designs are needed.

### 4.2. BDNF and Stroke

BDNF plays a critical role in the neuronal system by supporting neuron survival and contributing to neurogenesis. A decrease in serum BDNF levels was confirmed in stroke patients in two systematic reviews that included 26 RCTs and 62 case–control studies, respectively. Indeed, several studies have shown that BDNF levels between stroke patients and control subjects were significantly lower than in the control group. A recent study conducted on 94 subjects, 47 stroke patients, most of whom had a mild form, and 47 age- and gender-matched controls, showed that plasma concentrations of BDNF were 2.5-fold lower in acute stroke patients, although with large individual differences in growth factor levels, including stroke localization and genetic factors [60].

A key issue in studies of BDNF level measurement after stroke appears to be the time at which BDNF levels were measured. Poststroke BDNF fluctuation may affect the validity of BDNF as a biomarker for stroke diagnosis. A recent observational study by Tuwar et al. (2023) showed that BDNF levels were low 24 h after admission (mean 2.94 ng/mL), increased sharply after 25–48 h (mean 6.79 ng/mL), and then, gradually decreased to 2.77 ng/mL after 96 h [61]. This implies that comparisons of BDNF levels in stroke and healthy control subjects could vary considerably depending on when BDNF measurements are taken, although in the meta-analysis performed by Mojatabavi et al. (2022), the overall pattern of BDNF does not appear to increase or decrease over time [54].

From the general findings, a key role for specific BDNF polymorphisms emerged: stroke risk was lower for the GG genotype in both homozygous and dominant models because BDNF secretion was impaired for the A allele (met-BDNF). The Val66Met mutation moves the G allele to an A allele at position 196 of the BDNF gene and exchanges the amino acid valine with methionine at codon 66 (Val66Met or Met66Met) [10,62]. This gene mutation, common in humans (prevalence of 20–30% in heterozygotes and approximately 4% in homozygotes), results in lower BDNF production and circulating levels and in greater susceptibility to neurodegenerative disorders [63]. The Met66Met and Val66Met polymorphisms lead to impairments in intracellular trafficking and regulate secretion in neurons [64], reducing activity-dependent secretion and BDNF intracellular distribution by impairing BDNF intracellular trafficking [65], which impairs neuroplasticity, and thus, the recovery of neurological damage in patients with stroke, leading to worse outcomes [53].

As mentioned earlier, genetic factors may be partly responsible for variability in functional recovery in stroke patients and should be included in prognostic assessment [53], although conflicting results emerged in [52]. Future studies focusing on ethnicity should be conducted to investigate the role that BDNF genotype actually plays in stroke occurrence.

### 4.3. BDNF and Physical Activity after Stroke

Based on our findings, therapeutic approaches aimed at increasing BDNF levels appear to be a key element in poststroke recovery. High-intensity aerobic exercise or physical training performed either as a single session or with a specific training program, independent of the performance of exercise or routine physical therapy or rehabilitation, increases BDNF concentration in a short time. Conversely, it could be hypothesized that the restoration of BDNF concentration could promote neurogenesis.

In humans, the release of BDNF from the brain was observed at rest and increased as much as threefold during exercise. In both states, the brain generates 70–80% of circulating BDNF [66]. Several studies have shown that exercise has many benefits for the brain, improving cognitive function, blood flow, and resistance to injury, and increasing *BDNF* gene expression in the brain [67]. In clinical terms, high-intensity, short-term activity may be suitable to promote the BDNF response, and conversely, to contribute to brain health [68]. Our understanding of the duration of such positive effects, and especially of how long increased BDNF is maintained in stroke patients, should be deepened.

### 4.4. Limitations of the Included Studies

The overall quality of the retrieved systematic reviews raises some doubts. First, although PRISMA or MOOSE guidelines were provided by almost all the included studies, and all the included studies used great caution in their conclusions, some limitations arose regarding the strength of the evidence, probably because of the great heterogeneity of the primary studies and the not-always-clear procedures for studies selection. Second, apart from Aschroft et al. (2022), the included meta-analyses used the I_2_ value (the percentage of total variation between studies that was due to heterogeneity rather than chance) as the only discriminator for their meta-analytic model (fixed vs. random). This approach, although largely adopted in meta-analysis, has been discouraged because it is not an absolute measure of heterogeneity [69]. Third, although in some cases the reported I_2_ value was low, the correct interpretation might not be the result of an actual lack of heterogeneity between studies, but rather, the result of a very small number of studies. Finally, differences in the measurement of BDNF concentration (timing or methods available for BDNF measurements) prevented us from drawing strong conclusions.

### 4.5. Limitations of Our Study

First, all systematic reviews were included regardless of their risk of bias assessment. In addition, one integrative review was also included. Although the authors of the cited integrative review stated that it was difficult to conduct an adequate systematic review for the topic under study (BDNF and CVD), their approach seemed very similar to that used in systematic reviews. Our choice, although justified, could have led to different evidence on the role of BDNF in CVDs such as stroke. A more systematic analysis of the current evidence on the association between BDNF changes and diseases other than stroke should be performed. Second, although ROBIS is a very consistent tool, it was difficult to apply. Much methodological information was found in the Section 3 or Section 4 sections of the included reviews. Third, the Egger’s linear regression test for publication bias was adopted even when the total number of studies did not surpass 10 units, as recommended in the Cochrane guidelines.

## 5. Conclusions

In the current evidence, the large heterogeneity between studies and the lack of striking RCT studies conducted considering specific populations defined on the basis of ethnicity, age, or sex, should be rectified so that the complexity of BDNF in severe cardiovascular disease and/or comorbidities (e.g., depression) can be clarified. In stroke and heart failure, altered BDNF levels or the Val66Met genotype could be recommended as biomarkers for predicting CVD risk. Physical activity increases BDNF levels in the short term; more studies should be performed to investigate the role of physical activity in maintaining optimal BDNF levels. Currently, BDNF is shown to be an optimal biomarker for stroke. In fact, several studies have shown a direct association between altered BDNF levels and stroke. The value of BDNF as a biomarker for CVDs other than stroke is more complex. Although some studies have shown an association between certain cardiovascular diseases other than stroke and altered BDNF levels, the current evidence that BDNF is an important marker of disease should be strengthened. To this end, an important first step is to identify standardized approaches for BDNF measurement methods to allow more accurate comparability among different studies. In addition, longitudinal studies in the healthy population and in-depth assessments of BDNF levels in CVD patients, together with prospective evaluations of BDNF level changes after CVD, are needed to allow for the appropriate integration of BDNF measurements among CVD biomarkers.

## Figures and Tables

**Table 1:** Study characteristics.

Review (Year) Country	Review Aim	Included Studies	PICO or PECO	RoB
Ashcroft et al. (2022) Australian [49]	To identify the intensity and duration of exercise required to produce increases in BDNF concentrations poststroke	6 RCTs 1 pseudo-randomized trial 10 nonrandomized studies	Population: patients who had survived a stroke (≥18 years). Intervention: exercise intervention of any modality. Comparator: if applicable, no intervention. Outcome: BDNF levels.	RoB 2 and ROBIN-I
Bao et al. (2018) Canada [50]	To give a more precise and comprehensive estimation of the association between BDNF rs6265 and ischemic stroke	7 case–control studies	Population: patients with ischemic stroke and healthy patients. Exposure: polymorphism rs6265. Comparator: other polymorphisms. Outcome: association between BDNF polymorphism and ischemic stroke.	Pre-defined scale provided by Jiang et al. [56]
Halloway et al. (2020) USA [51]	To evaluate the role of BDNF in the risk and presence of serious cardiovascular conditions	1 controlled intervention, 1 before–after study without control group 13 case–control studies 20 observational cohort and cross-sectional studies	Population: patients with CVD. Exposure: BDNF concentrations or BNDF genotype. Comparator: none. Outcome: association between BDNF and CVD (BDNF as a result of CVD event or BDNF as a cause of CVD event).	Quality Assessment of Case–control Studies, Quality Assessment for Observational Cohort and Cross-Sectional Studies, Quality Assessment Tool for Before–After Studies with No Control Group
Karantali et al. (2021) Greece [52]	To study BDNF serum level’s potential role as a biomarker in predicting functional outcomes in acute stroke	26 RCTs	Population: patients with stroke. Exposure: BDNF concentrations. Comparator: none. Outcome: functional outcome.	RoB 2
Liu et al. (2021) China [53]	To explore the distribution of Val66Met polymorphism and evaluate the effects of different genotypes on stroke functional recovery	Not reported	Population: patients with stroke. Exposure: Val66Met polymorphism. Comparator: different genotype. Outcome: functional recovery after stroke.	Strengthening the Reporting of Genetic Association studies (STREGA)
Mojatabavi et al. (2022) Iran [54]	To find the relationship between the altered level of BDNF and stroke	62 case–control studies	Population: patients with stroke vs. healthy population. Intervention: none or exercise intervention of any modality. Comparator: if applicable, no intervention. Outcome: BDNF levels.	QUADAS-2
Shobeiri et al. (2022) Iran [55]	To synthesize the existing evidence to assess serum and plasma BDNF levels in ischemic heart disease patients.	9 cross-sectional studies	Population: patients with ischemic heart disease vs. healthy controls. Exposure: ischemic heart disease. Comparator: none. Outcome: BDNF levels.	Newcastle–Ottawa Quality Assessment Scale

PICO: P = patients, I = intervention, C = comparator, O = outcome; PECO: P = patients, E = exposure, C = comparator, O = outcome; RoB = risk of bias.

**Table 2:** Risk of bias (ROBIS).

Author	Domain 1	Domain 2	Domain 3	Domain 4	RoB in the Review
Ashcroft et al. (2022) [49]	LOW	LOW	LOW	UNCLEAR	LOW
Bao et al. (2018) [50]	HIGH	UNCLEAR	LOW	LOW	HIGH
Halloway et al. (2020) [51]	UNCLEAR	LOW	LOW	LOW	UNCLEAR
Karantali et al. (2021) [52]	LOW	UNCLEAR	UNCLEAR	UNCLEAR	HIGH
Liu et al. (2021) [53]	UNCLEAR	UNCLEAR	UNCLEAR	UNCLEAR	HIGH
Mojatabavi et al. (2022) [54]	LOW	LOW	LOW	LOW	LOW
Shobeiri et al. (2022) [55]	UNCLEAR	LOW	LOW	UNCLEAR	UNCLEAR

Domain 1: study eligibility criteria; Domain 2: identification and selection of studies; Domain 3: data collection and study appraisal; Domain 4: synthesis and findings. RoB: Risk Of Bias.

**Table 3:** BDNF levels and CVD risk.

Review (Year)	Included Studies	Authors’ Conclusions
Halloway et al. (2020) [51]	1 controlled intervention, 1 before–after study without a control group, 13 case–control studies, 20 observational cohort and cross-sectional studies	(1) Patients with heart failure had lower BDNF concentrations. (2) Patients with unstable angina and myocardial infarction had higher BDNF concentrations. (3) Lower BDNF levels are predictive of CVD. (4) Higher BDNF levels in healthy individuals appeared to be associated with increased cardiovascular risk, such as BMI or blood pressure. (5) Mixed findings for BDNF genotype due to different distributions of ethnicity and cardiovascular conditions.
Shobeiri et al. (2022) [55]	9 cross-sectional studies	Significantly lower BDNF serum and plasma concentrations in patients with ischemic heart disease regardless of BDNF measurement methods

**Table 4:** BDNF genotype and stroke.

Review (Year)	Studies	Authors’ Conclusions
Ashcroft et al. (2022) [49]	6 RCTs 1 pseudo-randomized trial 10 nonrandomized studies	(1) Lower risk of stroke for GG genotype in homozygous and dominant models. (2) Single session and program of high-intensity aerobic exercise increase BDNF concentration.
Bao et al. (2018) [50]	7 case–control studies	Lower ischemic stroke risk for GG genotype in homozygous and dominant models.
Liu et al. (2021) [53]	Not reported	(1) Caucasians have fewer A alleles compared with Asian patients. (2) Genetic factors may partly account for the variability in stroke functional recovery.
Halloway et al. (2020) [51]	2 studies	Lower serum BDNF levels associated with poorer functional status 90 days after stroke and higher risk of poor outcomes at 2 and 7 years.
Karantali et al. (2021) [52]	26 RCTs	Role of BDNF in functional outcomes in stroke patients is unclear.
Mojatabavi et al. (2022) [54]	62 case–control studies	(1) Lower BDNF concentrations in patients with stroke. (2) No significant association between BDNF levels and neuronal rehabilitation. (3) Positive effect of physical training on BDNF levels, regardless of performing exercise or routine physiotherapy or rehabilitation, immediately after the intervention. (4) No significant effect on BDNF levels measured in a delayed time.

## Data Availability

All data are reported in the manuscript.

## Supplementary Materials

The following supporting information can be downloaded at: https://www.mdpi.com/article/10.3390/life13122252/s1, Table S1: Studies’ search strategy details; Table S2: Meta-analysis details.
