# Supplementary material for: Brain–Heart Axis: Brain-Derived Neurotrophic Factor and Cardiovascular Disease—A Review of Systematic Reviews
Source: Life (Basel). 2023 Nov 25;13(12):2252. doi: 10.3390/life13122252 (PMC10744648; doi:10.3390/life13122252)
Supplement: Supplementary file 1 [file life-13-02252-s001.zip › life-2698312-supplementary.pdf]

Table S1 – Studies Search Strategy details

Review (Year) Country	Search strategy
**Ashcroft et al. (2022) Australian**	APA Thesaurus of Psychological Index Terms, CINAHL, Embase Classic+Embase, Medline, PubMed, SportDiscus, Web of Science Core Collection. No search start date. Last search date: 31 January 2022. Search terms: stroke, exercise, brain-derived neurotrophic factor. Hand searching: yes. Eligibility criteria: 1) Human study (≥ 18 years); 2) Diagnosis of stroke; (3) Delivery of exercise intervention of any modality; (4) Measurement of blood derived BDNF concentration; (5) Experimental or observational study
**Bao et al. (2018) Canada**	PubMed, Embase, Cochrane Central Register of Controlled Trials (CENTRAL), Chinese National Knowledge Infrastructure (CNKI), and Chinese Biomedical Literature Database (CBM). No search start date. Last search date: 9 July 2017. Search terms: ("ischemic stroke") and ("BDNF" or "brainderived neurotrophic factor") and ("polymorphism" or "mutation" or "variant" or "SNP" or "single nucleotide polymorphism"). Eligibility criteria: (1) case-control study; (2) studies demonstrated the association between BDNF polymorphism and ischemic stroke or post-stroke depression; (3) sufficient data withing the individual publication. Exclusion criteria: (1) repeat publications, abstracts, dissertations or reviews; (2) studies not meeting all of the inclusion criteria.
**Halloway et al. (2020) USA**	PubMed, Cumulative Index to Nursing and Allied Health Literature (CINAHL), and SciVerse Scopus. No search start date. Last search date: July 2019. Search terms: brain-derived neurotrophic factor (MeSH), BDNF (text word), trkB (MeSH), pro-BDNF (text word), and NTRK2 (text word), cardiovascular disease, coronary disease, heart failure, heart diseases, atrial fibrillation, myocardial infarction, cardiacmyocytes, and myocardial contractility. Eligibility criteria: (1) English language; (2) human subjectes; (3) BDNF measured as serum, plasma, or BDNF gene; (4) study involved risk or presence of serious cardiovascular conditions; (5) quantitative data analyses. Exclusion criteria: studies on comorbid condition (e.g. schizophrenia) not directly related to serious cardiovascular conditions or risk for serious cardiovascular conditions.
**Karantali et al. (2021) Greece**	PubMed, Web of Science Search engines, and the Cochrane database. No search start date. No last search date. Search terms: (stroke OR cerebrovascular disease OR CVD) AND (BDNF OR brain-derived neurotrophic factor OR neurotrophin). Eligibility criteria: (1) patients with stroke (ischemic or hemorrhagic) (2) BDNF values in serum, (3) acute phase (within 14 days after stroke), (4) participants 17 years old or older, male or female, (5) stroke severity, stroke volume infarct, and functional outcome as the primary or secondary of the study. Exclusion criteria: (1) animal studies, (2) review or

	meta-analysis, (3) study including below 5 participants, (4) study in healthy individuals.
**Liu et al. (2021) China**	PubMed, EMBASE, Ovid, Wiley Online Library as well as the Chinese databases of China National Knowledge Infrastructure (CNKI), WANFANG DATA, and China Science and Technology Journal Database (VIP). No search start date. Last search date: 4 May 2020. Search terms: BDNF, brain-derived neurotrophic factor, codon66, G196A, rs6265, Val66Met, stroke. Eligibility criteria: (1) studies focused on investigating the relationships between BDNF Val66Met and stroke and (2) articles providing the distribution of BDNF Val66Met genotypes in patients with stroke. Exclusion criteria: (1) reviews and animal studies; (2) articles focused on the BDNF serum level, not on BDNF gene polymorphisms; and (3) articles providing no distribution information of BDNF Val66Met polymorphisms.
**Mojatabavi et al. (2022) Iran**	PubMed, Web of Science and Scopus. No search start date. Last search date: 7 August 2021. Search terms: “stroke” OR “ischemic stroke” OR “hemorrhagic stroke” OR “Cerebrovascular Accident” OR “Brain Vascular Accident” AND “Brain-Derived Neurotrophic Factor” OR “BDNF”. Eligibility criteria: (1) a study measuring serum or plasma levels of BDNF in PwS. Studies (a) with animal subjects, (b) review or meta-analysis studies, (c) genetic investigation were excluded from the study (d) using other measuring methods rather than ELISA, like western blot, mass spectrometry or immunohistochemistry
**Shobeiri et al. (2022) Iran**	PubMed, Embase, Web of Science, and Cochrane Library. No search start date. Last search date: January 2022. Search terms: "BDNF" OR "brain-derived neurotrophic factor" AND "ischemic heart" OR "coronary artery disease" and other related terms. Eligibility criteria: (1) clinical studies with a control group that measured serum BDNF concentration in both control and IHD groups, (2) Cross-sectional studies, and interventional studies in which the BDNF concentration was measured before the intervention. Exclusion criteria: (1) case report or case series, (2) interventional study that reported the BDNF concentration postintervention; (3) letter, review, or conference abstract, (4) pre-clinical studies

Table S2 – Meta-analyses details

Studies reporting BDNF serum or plasma	No of studies (no of participants in analysis)	Study Types	Details of meta-analysis	Results
**Ashcroft et al. (2022) Australian**	17 (687)	6 RCT; 1 pseudo-randomzied trial, 10 non randomized studies	MD and 95%CI - Fixed and Random effects according to the following criteria: (1) clinically homogenous, and (2) statistically homogenous determined using a χ2 test (I2).	Single Session of Low-Intensity Aerobic Exercise: MD −0.04, [95% CI, −0.30 to 0.22], P=0.76, I2 = 0%; Single Session of Moderate-Intensity Aerobic Exercise: MD 0.04; [95% CI, −0.21 to 0.30]; P=0.73, I2 = 0%; Single Session of High-Intensity Aerobic Exercise: MD 2.49; [95% CI, 1.10–3.88]; P=0.001, I2 = 0%; Program of Low-Intensity Aerobic Exercise: MD 0.77; [95% CI, −0.83 to 2.38]; P=0.35, I2 = 0%; Program of Moderate-Intensity Aerobic Exercise: MD −0.22; [95% CI, −3.31 to 2.88]; P=0.89, I2 = 75%; Program of High-Intensity Aerobic Exercise: MD 3.42; [95% CI, 1.92–4.92]; P=0.00, , I2 = 2%; Effect of a Program of Nonaerobic Exercise: MD 0.72; [95% CI, −0.05 to 1.50]; P=0.07, , I2 = 90%
**Bao et al. (2018)**	7 (cases: 1287, 1032)	Case-control	OR 95%CI - Comparisons among genetic models such as homozygous (GG versus AA), heterozygous (GG versus GA), dominant (GG versus GA + AA), and allelic models (G versus A). I2 greater than 50% was considered to be with obvious heterogeneity, and the random-effects model (the DerSimonian and Laird method) was used for the metaanalysis. Otherwise, the fixed-effects model (the Mantel–Haenszel method) was used.	Lower risk of ischemic stroke for the GG genotype in the homozygous model and dominant model (GG versus AA, OR = .57, 95% CI = .43-0.75, P < .0001; GG versus GA + AA, OR = .80, 95% CI = .65-0.98, P = .03). Subgroup analysis by ethnicity: lower susceptibility of ischemic stroke in Asians in all genetic models except heterozygous (GG versus AA, OR = .58, 95% CI = .43-0.77, P=.0002; GG versus GA + AA, OR = .78, 95% CI = .62-0.97, P = .03; G versus A, OR = .77, 95% CI = .67-0.89, P = .0003).
**Liu et al. (2021)**	15 (No of pts = not reported)	Logitudinal studies	OR 95% CI - The fixed-effects model when the I2 was <50% and P was >.1; otherwise, otherwise the random-effects model. Egger's linear regression test for publication bias	The pooled OR(GA/GG) and OR(AA/GG) were 1.21 (95% CI: 0.95-1.55) and 2.45 (95% CI: 1.74-3.45). Dignificant difference in stroke functional recovery between the AA and GA+GG genotypes (OR = 1.90, 95% CI: 1.17-3.10, P = .010, I2 = 69.2%;). Sensitivity analysis: OR = 1.422; 95% CI: 1.07-1.90; P =.017.

Studies reporting BDNF serum or plasma	No of studies (no of participants in analysis)	Study Types	Details of meta-analysis	Results

**Mojatabavi et al. (2022) Iran**	16 (738 patients before and after training)	Pre-post studies	SMD and 95%CI - Fixed effects models if the results were homogeneous (I2 < 40% and p-value > 0.05). Random effect models (DerSimonian and Laird method) if results were heterogeneous (I2 ≥ 40% or p-value ≤ 0.05)	Immediate group (SMD = 0.49, 95%CI: 0.09 to 0.88), p-value = 0.02, I2 = 85%, p-value < 0.001). Exercise group BDNF levels significantly increased immediately after physical training (SMD = 0.75, 95%CI: 0.25 to 1.25, p-value = 0.003). No subgroup differences . Sensitivity analysis: (SMD = 0.34, 95%CI: − 0.03 to 0.71, I2 = 82%). Delayed group: no significant effect of the intervention (SMD: = 0.02, 95%CI: − 0.43 to 0.47, I2 = 83%). Patients with stroke had significantly lower serum BDNF levels than HC (SMD = − 1.02, 95%CI: − 1.47 to − 0.57, p-value < 0.001, I2 = 96%, p-value < 0.001). Sensitivity analysis: lower serum BDNF levels in patients with stroke than HC (SMD: − 0.92, 95%CI: − 1.35 to − 0.50, p-value < 0.001, I2 = 96%, p-value < 0.001). The age partially explained the high heterogeneity (correlation coefficient = − 0.11, R2 = 62.81%, p-value = 0.000).
**Karantali et al. (2021)**	6 (No. of patients not reported)	RCT	SMD and 95%CI - Fixed or random effects models according to the heterogeneity of the data. Egger's linear regression test for publication bias	Patients with acutte stroke had a significantly lower BDNF levele compared to controls (SMD: .2.37, 95%CI: -4.36, -0.38,). Sensitivity analysis results: SMD: -5.91, 95%CI: -8.40, -3.41, p < 0.0001)
**Shobeiri et al. (2022)**	9 (1137 cases, 724 controls)	Cross-sectional studies	SMD and 95%CI - Thresholds for heterogeneity (I2) were ≤ 25%, 26–75%, and ≤ 75% for low, moderate, and high heterogeneity. Random effect model (DerSimonian and Laird). Egger's linear regression test for publication bias	Insignificant lower levels of BDNF in IHD compared with controls (SMD = -0.57, 95% CI [-1.18; 0.04], p-value = 0.068). After removing outliers: SMD − 0.41 (95% CI [-0.78; -0.03], p-value = 0.03). Subgroup analysis: no significant difference between serum and plasma levels (p-value = 0.53). No predictor for the difference in BDNF levels.
